# The Foreign Journals: Anatomy and Physiology

**Published:** 1842-10

**Authors:** 


### PART THIRD.

### Selections from the British and Foreign Journals.

### I. THE FOREIGN JOURNALS.

### ANATOMY AND PHYSIOLOGY.

*On the minute Structure of the Spleen.* By M. Bourgery.

In an injected spleen the discernible anatomical elements of its structure are ten in number: 1, vesicular membranes; 2, blood-vessels; 3, floating vascular corpuscles; 4, a granulo-capillary basis; 5, a splenic liquid; 6, splenic glands, which are all lymphatic glands; 7, lymphatic vessels; 8, nerves; 9, cellular tissue; 10, a membrane enveloping the whole spleen. The first five of these compose the *vesicular apparatus* of the spleen, the blood-vessels being placed here because it is in these parts that they offer their most remarkable peculiarities. The sixth and seventh form the *glandular apparatus.* The last three are common to all parts of the spleen.

1. *Splenic vesicles.* They are distributed in every part, being separated by partitions, to whose surface their's is as 3·2. In the dried spleen they are irregularly polyhedral; in the injected spleen spheroid or ovoid, and this, it is probable, is their form during life, when they are filled by fluid. In the calf they measure, on an average, about $\frac{1}{8}$ of an inch in diameter; in the dog and sheep they are of similar size; in man they are smaller and more regular, their average diameter being about $\frac{1}{25}$ of an inch. None of them have a simple form; but the walls of all are traversed by vessels which, covered by the lining membrane, form folds or falciform or crescentic lamellæ, like those formed by the umbilical vessels behind the peritoneum. The result of these folds, of which there is a series of various extent, is to decompose the principal cavity into recesses, and these again into loculi, on the bases of which are seen in relief the glands and granules and capillary ramifications.

In these vessels there are two kinds of orifices: 1. Those by which all the vesicles communicate with one another, which are nearly circular, with thin edges covered by the lining membrane. There are two or three of these in each of the larger vesicles, and the diameter of each is about $\frac{1}{2}$ or $\frac{1}{4}$ of that of the whole vesicle. 2. The venous orifices, which are less numerous, and only scattered here and there, on any part of the walls of the vesicles indifferently. They are the absorbent mouths of veins of the same size as themselves, which are passing to the veins of the partitions; and they are guarded by a crescentic fold, which forms an incomplete valve.

The *intervesicular partitions,* or rather *spaces,* are formed by the separation of the enveloping membranes of adjacent vesicles, and contain the vessels and the splenic glands. Their width or thickness is inversely proportioned to the size of the vesicles. Also, where two vesicles only lie together the partitions are narrow; where several meet they are enlarged into irregular spaces filled with glands.

The enveloping membranes of the vesicles are continuous with one another throughout the spleen; so that one may consider them as forming a single membrane, homogeneous in every part, and divided into thousands of little ampullæ, isolated by constrictions, which constitute their orifices of communication, and supported by ramifications of vessels, which form a kind of soft frame-work of the whole organ. The membrane of each vesicle is formed of a single layer, from $\frac{1}{150}$ to $\frac{1}{300}$ of an inch thick; but its organization is very complex, for it contains the granulo-vascular basis with its close network of capillary blood-vessels and lymphatics. It cannot, therefore, be considered as produced by a mere dilatation of the common internal coat of the veins; though, as will presently appear, the veins of the spleen present characters exactly analogous to those of the vesicles.

II. *Blood-vessels.* These, according to the form, size, and distribution, may be described in three sets: 1, the large trunks or splenic vessels, commonly so called; 2, the intervesicular vessels; 3, the vesicular vessels. The *splenic vessels* pass, artery and vein together dividing three or four times, towards the periphery of the spleen. The veins are perforated by small, circular holes, which lead to the veins of the partitions or intervesicular veins. The terminal veins are decomposed, in the course of their canal, into a succession of cellules, separated by vascular constrictions, of which the organic composition is absolutely identical with that of the vesicles which succeed to them. The *intervesicular vessels,* arising from the preceding, run in the partitions, where they are distributed to the splenic glands and the vesicular membranes. In man they have a diameter of about $\frac{1}{500}$ of an inch. The vessels of the glands pass abruptly into them, like those of the corpus cavernosum. The *vesicular veins* form those little folds on the surface of the small cavities which have been already mentioned. But, by a singular arrangement, their branches project into the vesicular cavity, to distribute themselves to the floating corpuscles which are appended to their ultimate branches, like a bunch of grapes, according to the comparison of Malpighi. On the basis of the membrane the last capillaries form with the lymphatics the granulo-vascular network. As to the venules, there are two kinds of them: those of the common capillary network, and the *absorbent venules,* which are much larger, and which are seen to open by free orifices in the cavities of the vesicles. Lastly, all the small vessels of the spleen, the intervesicular as well as the vesicular, are distinguished, when turgid, by continuous series of dilatations and constrictions, which give them a remarkably knotted aspect.

III. *Floating vascular corpuscles.* These float within the vesicles, appended, as in pedicles, to the last branches of the capillary blood-vessels and lymphatics. They are formed of a lenticular nucleus, from which, when they are turgid, there project the stems of little aigrettes, radiating towards the circumference, so as to resemble the flower of an umbelliferous plant. These aigrettes themselves are composed of a filament, terminated by brilliant little spherules, collected in the form of a chaplet. The corpuscular nuclei have, in the calf, a diameter of from $\frac{1}{350}$ to $\frac{1}{50}$ of an inch, and their capillaries are from $\frac{1}{1200}$ to $\frac{1}{2500}$ of an inch in diameter. In man the corpuscles are from $\frac{1}{500}$ to $\frac{1}{250}$ of an inch in diameter, and the capillaries which pass to or from them have a caliber varying from $\frac{1}{800}$ to $\frac{1}{3000}$ of an inch.

IV. *Granulo-capillary basis* is the name given by M. Bourgery, on account of its aspect, to the membrane of the vesicles, which is itself composed of two elements: 1st, spherical granules, which are very pale, juxtaposed, and from $\frac{1}{800}$ to $\frac{1}{600}$ of an inch in diameter; and 2dly, arterial, venous, and lymphatic capillaries, varying from $\frac{1}{2500}$ to $\frac{1}{5000}$ or $\frac{1}{7000}$ of an inch in diameter, distinct from the larger capillaries of the floating corpuscles. The vascular net forms a lacework several layers thick.

V. *Splenic liquid.* This liquid or *splenic blood* appears to be the product of an elaboration by the floating corpuscles and the granulo-capillary basis, which is deposited in the cavities of the vesicles, and is taken up again by the absorbent vessels of their walls. It is thick, viscid, and brownish red; and, under the microscope, seems to be composed of several kinds of globules suspended in a yellowish, unctuous liquid; especially of, 1st, *lenticular* globules, some of which are surrounded by a red limbus, and do not appear to differ from ordinary blood-globules, while others are colourless; and, 2dly, whitish globules, irregular in form and size, which remind one of those found in the chyle and lymph.

VI. *Splenic glands.* The preceding parts compose the vesicular portion of the spleen, the two next form its glandular apparatus. These glands constitute, in respect of volume and consistence, the most considerable organic element of the spleen. United by cords of the same substance, they fill up, with the ramifications of the vessels, the intervesicular partitions or spaces. Their greatest diameter, when in a state of repletion, is, in the calf, about $\frac{1}{25}$ of an inch, in man about $\frac{1}{100}$. In the ox they are as much as $\frac{1}{12}$ of an inch, or more, in diameter, and one can see them with the naked eye, in the form of brown or whitish corpuscles. It is these which most authors have regarded as the vesicular glandules of Malpighi. They are isolated or agglomerated in the intervesicular spaces, and are united by cords into chaplets which extend through every part of the spleen. They receive a great number of capillaries, and a thin layer of their substance well injected seems to resolve itself into infinitely small granules and capillaries.

The relation which the lymphatic vessels bear to these glands shows their physiological import. One sees plexuses of lymphatic vessels interlaced on their surface, afferent trunks penetrating them and their connecting cords, efferent trunks leaving them and proceeding to the intervesicular blood-vessels, and infinite subdivisions of the minute lymphatics in the interior of both them and their cords. So that there can be no doubt that these splenic glands are merely microscopic lymphatic glands.

VII. *Lymphatic vessels.* These exist in immense number in the spleen. Arising from the granulo-capillary network, in which they form the very closest lacework, they join to themselves the branches from the floating corpuscles, and with these they pass to the intervesicular glands. There are from fifteen to twenty of their larger branches on each vesicle, and the form of these is peculiar. Externally each large branch is surrounded by numerous interlacing small ones, just as the blood-vessels of the digestive organs are surrounded by lymphatics of larger size. And internally, besides possessing very distinct valves, they are divided into loculi, the agglomeration of which at their junctions gives the notion of a kind of rudimental gland: as if the lymphatic vessels were not merely canals of transport, but organs of elaboration.

VIII. On the remaining three elements M. Bourgery offers no peculiar remarks. Neither does he give any definite explanation of his view of the office of the spleen; he only calls it "a vast lymphatico-sanguine gland," regarding the veins and the vesicles as apparatus for the elaboration of blood, and the glands and their connecting cord as one great lymphatic gland, broken up into microscopic glands, united by cords of the same substance that it may extend itself through every part of the spleen, and everywhere surround the vesicles.

*Gazette Médicale. Juin* 11, 1842.

(*From a memoir read before the Academy of Sciences*, 6*th June.*)

---

*Fatty and Cellular Degeneration of Muscle.* By Dr. JOSEPH ENGEL.

ACCORDING to Gluge, the fatty degeneration of muscle consists in the deposition of fat, partly in a free, partly in an encysted state, between the primitive muscular fibres. The author is of opinion that the above is not the only metamorphosis which takes place, but is not able to persuade himself that an actual transformation of muscular into cellular tissue is to be admitted. So long as muscle, examined by the microscope, undergoes no other alteration than a mere change of colour, the form of the primary and secondary muscular bundles remains unaltered, except that their sheaths appear covered with a greater number of granulations, the greater part of which, however, are readily removed by a slight pressure. The sheath itself of the fibre appears more brittle and easily ruptured than before; the least pressure destroys its continuity. In the same proportion in which the fatty condition of the muscle and its loss of colour become apparent to the naked eye, do the longitudinal lines of the muscular bundles become more evident, the granulations more numerous, and the bundles themselves appear covered with a multitude of large bladders of fat, which, from becoming flattened, present various forms, and are at length united, partly with each other, partly with the bundles of muscular fibres by a tolerably adhesive substance. The author failed to detect fat in a free state in fresh muscle; which, however, if treated with spirit of wine, gives out fat in notable quantity. Yet in this case the fat seems to be simply extracted from the sebaceous bladders or cysts by the action of the spirit of wine; these cysts, thus deprived of their contents, being visible, in a shrivelled state, between the bundles of muscular fibre. Water causes the fat to solidify, and to appear surrounding the muscular bundles like a sheath, from among which, by a slight pressure, it may be forced in the shape of half cylinders. The muscular bundles amid this deposition of fat, partly free, and partly encysted, lose entirely their sheaths; so that their delicate fibrillæ (primitive fibres,) after the fat being washed away, lie bare. This condition, and no other, it is which constitutes the so-called degeneration of muscle into fatty and cellular tissue, and which consists of nothing but a deposition of fat between the bundles of muscular fibre deprived of their sheaths. The sheath of the muscular bundles appears to be indispensable to elasticity, firmness, equal action, and vital function of muscle. Whether the change of colour which supervenes after some time in separated muscle be owing to the loss of the sheath, the author is not prepared to say. A question arises, whether the destruction of the sheath of the muscular bundles be owing to the deposition of fat, or whether the converse relation be the real one, or finally whether both changes be synchronous results of a common cause.

*Oesterreich. Medicinische Wochenschrift*, No. ix. den xxvi. *Februar*, 1842.

---

*Pathology of the Tissues.* By Dr. JOSEPH ENGEL.

1. AN osteophysis, or formation of bone on the inner surface of the skull, in the case of pregnant women, is a frequent occurrence in Austria. It has hence been inferred that there is a certain relation between pregnancy and this species of osteous growth; although against the supposition that there is any such necessary connexion militates the fact that in England this growth has never been observed in pregnant women, and that it has been noticed in women who were never pregnant, and also in men.

The first appreciable phenomenon in this peculiar morbid process is the appearance of a gelatinous, yellowish-red exudation on the outer surface of the dura mater, without any apparent actual implication of that membrane. This exudation, examined by the microscope, exhibits cells containing nuclei, which become united by an amorphous, gluey substance, and extend themselves on one or on both sides by fibre-like prolongations. The nucleus of the cell is tolerably large, and is sometimes surrounded by a capsule. The dura mater becomes adherent, in consequence of this exudation, to the corresponding part of the cranial bone; and from this point dates the deposition of earthy salts, which is effected by the same process by which, in cases of fracture, the formation of bone takes place from the deposition of callus. The phenomena are analogous to those which take place in periostitis, in which exudation from the bone amalgamates with phosphate of lime, resulting in osteophysis.

The frequency of this morbid affection and the circumstance of its occurring in advanced life, are additional grounds for regarding it as having no necessary connexion with the pregnant state. Rarely does this disease manifest itself, during life, by any symptoms.

2. *Proneness of articular cartilage to suppuration.* The author illustrates this proposition by the case of a phthisical subject in whom the knee-joint exhibited all the appearances of chronic inflammation. The synovial membrane was connected with the subjacent fat by a glairy, yellow exudation, and was thickened though not injected; the cavity of the capsule was filled with healthy pus, a thin layer of which attached itself to the surface of the capsule; the semilunar cartilages were entire; the crucial ligaments very friable; the cartilaginous extremities of the femur and tibia were very thin, sharp, and their edges easily separable from the subjacent bone, and divisible into three layers. The brittleness of these "pathological cartilages" was remarkable. They were easily pressed, under the microscope, into an homogeneous, granular mass.

3. *The mode of cicatrization of typhus ulcers.* After the ulcerative process is at an end, and the so-called typhus ulcer appears clean, its bottom, situated in the cellular or muscular tissue, appears, when viewed under an oblique light, covered with an exceedingly fine and remarkable cuticle. This begins from the abrupt margin of the usually slate-gray-coloured ulcer, and runs towards the centre of the ulcer, but does not entirely reach or cover this point. The membranous expansion which extends over the rest of the ulcer, is partly of a cellular, partly of a fibrous character, separate or mixed. The cells are round or elliptical, and seem to form fibres by mutual approximation of their homologous sides and the subsequent absorption of the walls of the cells where these are in contact with each other. In other cases fibrous bundles are observable, which appear to be prolongations from a single cell. The author's observations do not permit him to say positively whether this last species of fibrous formation is the origin of submucous cellular tissue, the former of the mucous membrane itself.

4. *Morbid deposits on the serous membranes in cases of protracted diarrhœa.* Every anatomist knows that viscid, thin, colourless exudation which appears as a layer on the free surface of the great serous membranes, in cases of exhausting diarrhœa. In children, more especially, this exudation is not a rare occurrence, and in the most of this class of cases is limited to the pulmonary pleura. The false membrane is easily obtained by stripping it off, though seldom in such purity and quantity as to allow an analysis to be made of it. Examined with the microscope, it appears an amorphous, gluey mass, amid which are discernible numerous round cells, one or both of the sides of which are prolonged into threads; the cells seem two or three times larger than blood-corpuscles, and appear filled with many pigmentous nucleoli. In many there was discernible a pale, round granule or nucleus, about the size of a blood-corpuscle; and this nucleus seemed sometimes made up of many granules. The ultimate constitution of this false membrane is consequently not easily determined.

*Oesterreich. Medicinische Wochenschrift*, No. iii. *Jänuar*, 1842.

---

### *Pathological Observations confirming the Experiments of Panizza on the Nerves of the Tongue.*

In this paper, which seems to be published by the Medico-Chirurgical Society of Bologna, by whom the patient was examined, there is but one original observation. It relates to a man fifty-two years old, who, after suffering for some time from wandering pains in his limbs, and being actively treated for them, was attacked by pain in the head and dulness of sensation in the left side of the face. The latter slowly extended over the right side also, and to the interior of the face, and ended in complete insensibility of every part supplied by the fifth nerves. Sight, hearing, and the sense of smell were unaffected. Many experiments were made to determine his power of taste; and it was found perfect for all sapid substances: he could distinguish the qualities of each article of food at his meals as well as he could when in health, and could discern the change and loss of savour which ensued when he kept them in his mouth. From the loss of common sensation, however, swallowing was difficult: he could not drink a large quantity at a time, and he was often choked in the attempt to swallow fluids. He was treated with electricity with slight temporary advantage. He died of intercurrent disease, but his body was not examined.

*Bulletino della Scienze Mediche. Aprile,* 1841.

---

### *On the minute Structure of the Lungs in Man and the Mammalia.* By M. Bourgery.

The peculiarities of M. Bourgery's views relate chiefly to the mode in which the minutest bronchial tubes terminate. "Each lobule," he says, "receives a single central bronchial branch, which forms the common tree of its air-tubes; or, if the lobule be large, two or even three of these branches may enter into it. The smallest of these lose themselves laterally in the manner presently to be described; a single tube, which is the continuation of the trunk, reaches the peripheral base of the lobule, and there ramifying, turns towards one of its angles, which forms the terminal summit. Proceeding from this decreasing central tree there arise from every side, in alternate succession and in star-like radiation, secondary branches, which I have named *bronchial ramified canals,* the ultimate expansion of the tracheal tree, beyond which the *labyrinthic* arrangement commences."

This labyrinthic arrangement is made up of numberless minute canals, (*aerial labyrinthic canals,*) arising from the bronchial ramified canals, running tortuously in every direction, and branching and anastomosing in the most multiform manner. They thus inclose numberless irregular spaces, in which, as well as on their walls, the blood-vessels ramify and form their capillary network. They give the idea of a space all divided with thousands of tortuous branchings, everywhere continuous with itself, and where there is no termination except the one common orifice of entry and exit in the trunk of the bronchial tree of each lobule. In no observed instance was there anything like a cul-de-sac or cæcal termination of any of the canals; "whatever point or surface he observed, there are flexuous canals anastomosing in every plane, but nowhere are there any straight canals without anastomoses or any vesicles." The sinuous canals, moreover, are from space to space constricted, not, as Willis supposed by ligamentous fibres, but by annular vessels, circumscribing in their intervals *loculi,* at the bases of which are the orifices of other labyrinthic canals, and giving that appearance of chains of cellules which forms the basis of the theories of Malpighi and Helvetius. In such canals as these, the ramified bronchial canals, after giving off great numbers of them from their sides, ultimately, by dividing twice or three times, terminate; and their terminal branches enter into the common labyrinth, comporting themselves like the tubes given off from their sides.

*Gazette Médicale. Juillet* 16, 1842.
(*From a memoir read at the Institute, July* 11, 1842.)

---

### *On the Muscular Coat of the Stomach.* By M. Noel Gueneau de Mussy.

The author makes four layers of muscular fibres in the stomach, by dividing the superficial or longitudinal layer into two, which he calls, respectively, cardiac and pyloric. The former he regards as the continuation of the longitudinal fibres of the œsophagus, the latter of those of the duodenum. [The distinction is unimportant, and not accordant with the analogy of the muscular coat of the stomach to that of the œsophagus and intestines, which is exact, if the stomach be regarded as a part of the one tube dilated on its left side. The arrangement of its muscular fibres is the same as would be produced in any other part of the canal, if such a dilatation could be effected.]

*Gazette Médicale. Juin* 4, 1842.

*On the Development of the Entozoa.* By M. Mandl.

In support of the doctrine of the spontaneous generation of the entozoa, the frequent occurrence of the ascaris nigro-venosus in the lungs of frogs has often been quoted. M. Mandl has succeeded, by a microscope magnifying 250 diameters, in detecting the coloured eggs of these entozoa in the lungs of frogs in which no trace of the entozoa themselves could be discerned. This, he observes, makes it very probable that these little ova, the diameter of which is scarcely more than four-times that of the blood-corpuscle of the same animal, have been carried into the lungs either in respiration or by some other passage. [The conclusion is in no degree warranted by the premises.]

*Gazette Médicale. Juillet* 2, 1842.

